# Image based fog density estimation

**DOI:** 10.1371/journal.pone.0323536

**Published:** 2025-06-02

**Authors:** Mingrui Dai, Weifeng Shi, Guohua Li

**Affiliations:** Institute of Computing Technology, China Academy of Railway Sciences Corporation Limited, Beijing, China; Guangdong University of Petrochemical Technology, CHINA

## Abstract

Although the application of image-based fog density estimation brings excellent convenience and low-cost methods, the accuracy of such methods still needs to be improved, and further research is encouraged on accuracy evaluation methods. To improve the accuracy and computational efficiency of fog density estimation in images, we first construct three image features based on the image dark channel information, the image saturation information, and the proportion of gray noise points, respectively. Then, we use a feature fusion method to estimate fog density in the images. In addition, two indicators have been constructed to evaluate the accuracy of various fog density estimation methods. These two indicators are the sequential error indicator and the proportional error indicator, which are calculated using fog image sequences with known density values. These two new indicators enable the evaluation of any fog density estimation method in terms of the ability to maintain order and ratio values. The experimental results show that the proposed method can effectively estimate the fog densities of images and display the best performance among the eight latest image-based methods for estimating fog density; the three features used in the proposed method significantly impact the effectiveness of image-based fog density estimation. The proposed method has been illustrated for fog density analysis of indoor and outdoor surveillance videos. The source code is available at https://github.com/Dai-MR/ImageFogDensityEsitmation.

## 1. Introduction

The measurement of fog density is an ancient and crucial problem because fog is one of the main factors affecting visibility [1–3]. Traditionally, visibility measurement is done through professional sensing equipment [4–6], which costs relatively high and is inconvenient. With the development of computer vision technology and artificial intelligence technology in recent years, estimating fog density through images has received increasing attention from researchers [7–9].

Based on the output data type, the existing image-based fog density estimation methods can be broadly categorized into two significant groups: (1) fog density level and (2) fog density value. The former is typically represented using levels such as fog-free, light fog, moderate fog, and heavy fog. The latter, fog density value, is a continuous non-negative real number, providing valuable insights into fog change characteristics and trends.

Significant progress has been made in estimating fog density levels. In 2018, M. I. Anwar et al. [9] presented a method for estimating the fog density level using a Support Vector Machine (SVM), classifying the synthetic data into homogeneous and heterogeneous fog. In 2019, Y. Chen et al. [10] proposed a fog density estimation algorithm using multiple features, including color, edge gradient, and transmittance. Their classification is completed by training a support vector machine classifier. Their experimental data are three levels: fog-free images, thin fog images, and dense fog images. In 2020, J. Dong et al. [11] built a model based on four features: color, dark channel, image entropy, and contrast. They used a multi-classification algorithm, S-DAGSVM, to classify fog images into four density levels: fog-free, light fog, medium fog, and dense fog. In 2023, W. Yang et al. [12] proposed a deep learning framework named VENet based on multi-visual feature fusion for fog visibility estimation. Their method comprises two subtask networks for fog level classification and fog visibility estimation, respectively. They employed a special feature extractor and an anchor-based regression method (ARM) to improve the accuracy. Five fog levels, including fog-free, low fog, medium fog, high fog, and dense fog, were classified in their experiments.

Besides machine learning methods, some researchers estimate the image fog level based on physics, mathematics, and statistics to avoid black box trouble from deep learning and lack of high-quality training data. For example, applying image entropy for fog density analysis and constructing dehazing algorithms [13] is common and effective. In 2023, R. Cao et al. [14] proposed an image-based method, denoted by GDEn, to estimate fog density levels. The method improved the accuracy and efficiency of analyzing acceptable meteorological conditions and validating fog density predictions. They used two types of image entropy: a two-dimensional directional entropy derived from four-direction Sobel operators and a combined entropy that integrates the image directional entropy and grayscale entropy.

Unlike fog density levels, the range of fog density values is non-negative continuous real numbers, which is not a simple classification issue. The main idea of estimating the image fog density value is establishing a mapping function or a model between the image fog density and the image features. For example, a referenceless perceptual fog density prediction model, called Fog Aware Density Evaluator (FADE), based on natural scene statistics and fog-aware statistical features, was proposed by L. K. Choi in 2015 [15]. FADE predicts the visibility of a foggy scene from a single image, which does not depend on the reference to a corresponding fog-free image, the salient objects in a scene, the side geographical camera information, the estimation of the depth-dependent transmission map, and the training on human-rated judgments. FADE only uses measurable deviations from statistical regularities observed in natural foggy and fog-free images. In 2018, Z. Ling et al. [16] developed a simple fog density evaluator (SFDE) by adopting a linear combination of three fog-relevant statistical features: the chroma variance, average saturation, and a Weber luminance contrast. These three features were selected by analyzing thirteen features of the image. In order to estimate fog density correctly and to remove fog from foggy images appropriately, a surrogate model for optical depth was presented by Y. Jiang et al. in 2017[17]. We name this model JSVC because it consists of three image features: dark-channel map (denoted by Jdark) proposed by K. He [18], saturation value of an image in HSV format, and chroma in the CIELab color space.

For the convenience of expression, researchers usually construct an index representing fog density. For example, in 2021, D. Ngo et al. [19] built an objective function to maximize the image’s saturation, brightness, and sharpness while minimizing the dark channel. We name this method HDE. In 2022, Guo H. et al. [20] proposed a fog density index defined as a function of the dark channel information and the pseudo-edge details information of the images (JdEg). As an application, both HDE and JdEg can be used to evaluate the effectiveness of dehazing algorithms. In 2024, S.-H. Hwang et al. [21] proposed an algorithm (AEA-RDCP) for estimating the image fog density, which uses the improved Dark Channel image (Jdark) to build the fog density function. Their creativity also includes the recognition and processing of sky regions.

These methods based on mechanism all use several practical image features. We list these representative features in Table 1 for convenient comparison. From the table, Jdark and saturation are the most commonly used features. Taking inspiration from this observation, we introduce both Jdark and saturation features in our model.

**Table 1:** Eight typical methods for estimating fog density and several features are used in calculating fog density.

Year	Method	Jdark	HSV.S	HSV.V	Chroma	Edge	Other
2015	FADE[15]	y	y			y	y
2017	JSVC[17]	y	y	y	y		
2018	SFDE[16]		y		y		y
2021	HDE[19]	y					y
2022	JdEg[20]	y				y	
2023	GDEn[14]					y	y
2024	AEA-RDCP[21]	y					

*Jdark is the information of the dark-channel map of the image.

*HSV.S is the S channel (saturation) value of the image in HSV format.

*HSV.V is the V channel value of an image in HSV format.

*The symbol “y” means the image feature (column) is used by this method (row).

In practice, capturing image sequences with different densities of fog accompanied by density values in the same scene has high time and capital costs. Therefore, constructing a synthetic fog image dataset is beneficial for evaluating the accuracy of the estimated fog density. Six years ago, M. Cordts et al. [22] and C. Sakaridis et al. [23] proposed a fog simulation approach on natural scenes, which leverages the semantic annotation of the scene as input to a novel dual-reference cross-bilaterally and applied it to the Cityscapes dataset. In 2024, Y. Xie et al. [24] introduced an end-to-end simulation method to generate foggy images. They constructed a new synthetic fog dataset named SynFog, which has three fog density levels. However, almost no literature quantitatively proposes using these known density information data to evaluate the accuracy of those existing fog density estimation methods.

Accuracy assessment is an important component of fog density estimation tasks. By combining physical instruments for fog density analysis, the estimation results of fog density will be more reliable. In 2007, K. Mori et al. [2] proposed a method of judging fog density using in-vehicle camera images and millimeter-wave (mm-W) radar data. This method determines fog density by evaluating both the visibility of a preceding vehicle and the distance to it. Their experimental results showed that judgments made by the proposed method achieve a precision rate of 85% when compared to the ground truth obtained by human judgments.

Considering that the existing methods for estimating fog density have some shortcomings [25], we in this paper propose a novel image fog density estimation method to improve the accuracy of fog density estimation and build two new indexes to evaluate the accuracy of the fog density estimation method. Our research highlights three aspects.

- We propose a method for estimating fog density using images. The new method is based on three significantly influential image features selected according to the analysis of existing methods (Table 1).
- Our method estimates fog density within an interval of [0,1], which aligns with general understanding and facilitates comparison of fog density in different scenes. Note that GDEn [14], FADE [15], and SFDE [16] methods do not limit the estimated fog density values to between 0 and 1.
- We present two indicators, the sequential error indicator, and the proportional error indicator, to evaluate the accuracy of fog density estimation results by comparing the known density values of the fog image sequences. These two indicators evaluate the accuracy of the estimation method from the perspectives of preserving order and preserving ratio value, respectively.

## 2. Data and methods

Firstly, we introduce the data we intend to analyze. Then, the proposed algorithm is described in Subsection 2.2. Finally, we propose two indicators for evaluating the error of fog density estimation results.

### 2.1. Data

In the next section, we tested and analyzed four datasets. Two are composed of synthesized fog images, which we use to validate the accuracy and performance of the proposed method. The other two are composed of images captured through surveillance videos from two actual traffic scenarios, which we use to demonstrate the application examples of our method.

#### 2.1.1. The color hazy image database.

**Fig 1:**
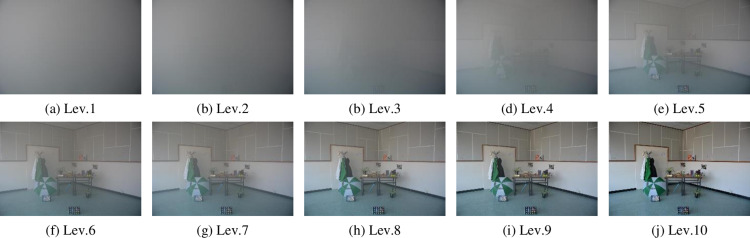
Ten images in different fog density levels in Scene 1 of CHIC. The fog density in the images from subfigure (a) to subfigure (j) continues to decrease. Lev.1 is the heaviest fog density, and Lev.10 is the fog-free.

The Color Hazy Image Database (CHIC, http://chic.u-bourgogne.fr/chicpage.php, accessed on 16 August 2022) was opened by El Khoury et al. [26,27]. This dataset comprises two categories, CHIC_Static_scenes and CHIC_Dynamic_scenes. In CHIC_Static_scenes, there are two indoor scenes, named Scene1 and Scene2. The images of the synthesized fog in both scenes were completed in a controllable environment. Each scene consists of 10 images with different fog densities, from heavy fog (Level 1) to fog-free (Level 10). As an example, ten images of Scene1 are displayed in Fig 1. In our experiment, we use the resized images of Scene1 and Scene2, and the size of each image is 1800 × 1200.

#### 2.1.2. The cityscapes dataset.

**Fig 2:**
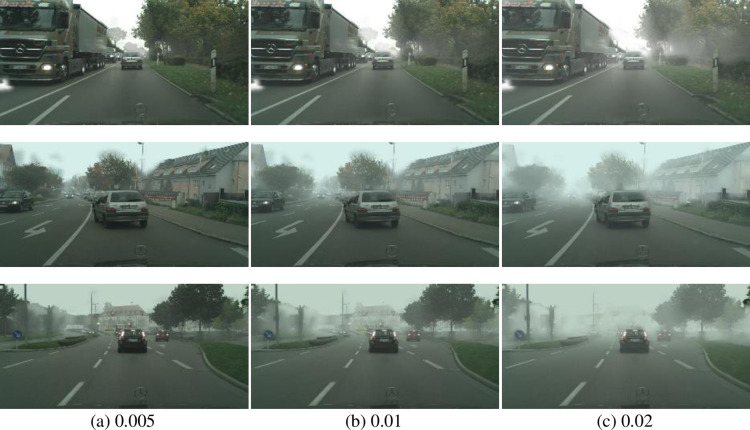
Nine sample images (three images with three fog density levels: 0.005, 0.01, 0.02) from the “lindau” database in the Cityscapes dataset.

The second experimental dataset we used is “lindau”, which is a subset of the cityscapes dataset, named Foggy Zurich, (https://people.ee.ethz.ch/~csakarid/SFSU_synthetic/, accessed on 5 April 2023) [22,23]. The “lindau” dataset consists of 177 images, of which 59 images are covered with shallow fog, 59 with moderate fog, and 59 with heavy fog. Fig 2 displays nine sample images from three different street scenes. Each row is a street scene with different fog densities, 0.005, 0.01, and 0.02.

#### 2.1.3. The key frames of highway monitoring.

**Fig 3:**
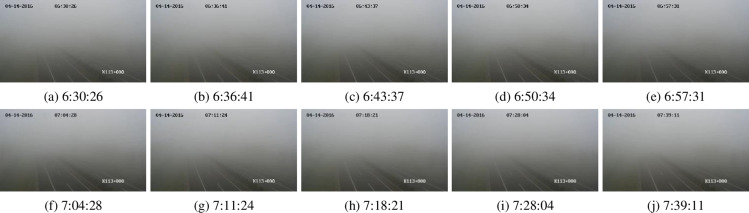
Sample ten keyframes abstracted from a highway monitoring video.

The third data is video surveillance data of an actual traffic scene from the fourth question in the 2020 China Post-Graduate Mathematical Contest in Modeling. The data can be downloaded from the website (https://cpipc.acge.org.cn/cw/hp/4, accessed on 16 March 2024). One hundred frames in the BMP image format captured from a highway surveillance video are offered. The size of each image is 1280 × 720. The data collection time is from 6:30:26–7:39:11 in the morning, and this is a foggy morning. Fig 3 displays nine sample frames of the dataset. The time intervals of these nine frames are roughly the same and include the first and last frames.

#### 2.1.4. The keyframes of airport monitoring video.

**Fig 4:**
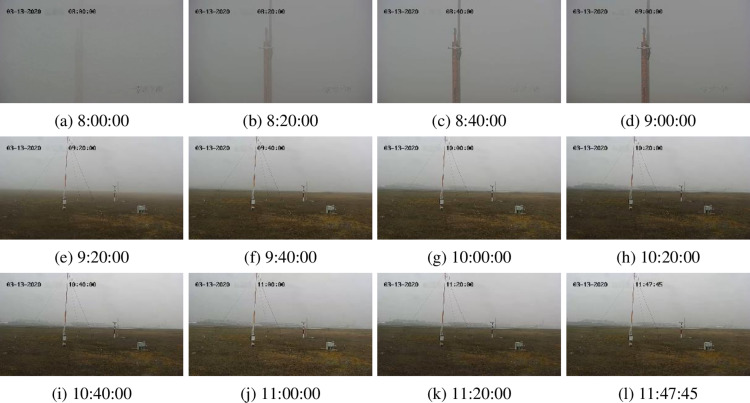
Sample 12 keyframes abstracted from an airport monitoring video.

The fourth data, Airport Video, is also from the fourth question in the 2020 China Post-Graduate Mathematical Contest in Modeling and can be downloaded from the website (https://cpipc.acge.org.cn/cw/ hp/4, accessed on 16-03-2024). The video was captured by a fixed-point rotatable camera and recorded an airport scene from 00:00:26–11:47:45 on March 13 (Friday), 2020. Considering that the fog density does not change much at night, we adopt a piece of video from the morning (8:00:00–11:47:45) for fog density analysis. It is a foggy morning. The size of each frame is 1280 × 720. The default play speed of the video is 25 frames per second. Fig 4 displays 12 key frames of the video. The time interval of these 14 frames is about 20 minutes, a remarkable time for displaying some remarkable changes in frames. From these subfigures, it can be found that the density of fog gradually decreases, and the visibility gradually improves.

### 2.2. The proposed method

**Fig 5:**
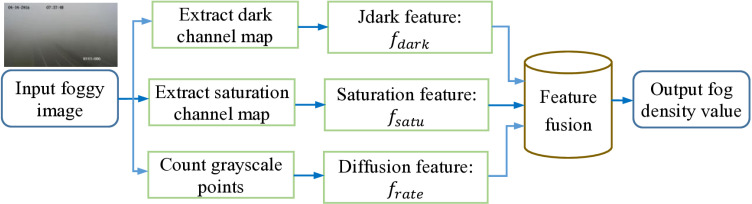
The proposed JSFD algorithm flowchart.

In this subsection, we propose a method to estimate the fog density of an image by fusing three new image features. The three features are built using the image dark channel information (Jdark), the image saturation information, and the proportion of gray noise points in the image. The first two features are the most effective features discovered based on state-of-the-art (SOTA) analysis, as shown in Table 1, while the last one is constructed to reflect the degree of fog diffusion. Our algorithm, named Ours or JSFD according to the three features, consists of five steps: calculating the Jdark feature, calculating the adjusted saturation feature, constructing the image’s grayscale points diffusion feature, feature fusion, and then outputting the estimated fog density of the image. The proposed JSFD algorithm flowchart is shown in Fig 5.

#### 2.2.1. Calculating Jdark feature.

The first step is to obtain the Jdark map by employing the dark channel prior (DCP) method [18] and constructing the adjusted Jdark feature $f_{dark}$. Referring to the DCP theory, the Jdark map ($J_{dark}$) can be obtained using an input image Iin the RGB (color) format as


$$J_{dark}(x)=\Gamma \left(\min_{y\in U(x)}\left(\min_{c\in \{R,G,B\}}I^{c}(y)\right)\right)$$ (1)

where $I^{c}$ is the *c* color channel of image *I*; *x* is a pixel of *I*, U(x) is a local patch centered at *x*, and *y* is the neighbor pixel around *x* in the patch. In addition, Γ(M) is the bidirectional filtering to matrix *M*, which improves the robustness of the Jdark map.

Different from the existing method, which calculates the Jdark feature using the simple average of all gray values in Jdark map, our Jdark feature $f_{dark}$ is constructed using those gray values no more than a threshold $\delta_{d}$. This parameter is an experimental value. We set $\delta_{d}=$0.85 as the default value considering the proportion of the area occupied by the sky and white objects in the image. This method still works even if the image does not contain a sky patch, as it only removes a small patch from the image. In this way, the index $f_{dark}$ can be obtained as


$$f_{dark}=\frac{1}{N}\sum xJ_{dark}(x)\chi (x),$$ (2)

where *N* is the number of non-zero elements (χ(x)=1which have been defined by an indicator function χ(x) defined as


$$\chi (x)=\left\{ \begin{array}{c} 0,J_{dark}(x)\geq \delta_{d}\\ 1,J_{dark}(x)<\delta_{d} \end{array}\right.$$ (3)

The main purpose of Eq (3) is to reduce the impact of white regions on fog density estimation, since the white regions have a significant impact on estimating fog density [20].

#### 2.2.2. Calculating adjusted saturation feature.

The second step is to calculate the saturation feature of the image by convert image *I* from RGB to HSV format and then constructing adjusted saturation feature $f_{satu}$.

Based on [28], the saturation of the image can be calculated as


$$S(x)=\frac{\max_{c\in \{R,G,B\}}I^{c}(x)-\min_{c\in \{R,G,B\}}I^{c}(x)}{\max_{c\in \{R,G,B\}}I^{c}(x)},$$ (4)

Note that according to [17], the relationship between the *H* channel and the density change of fog is not significant, so the *H* channel feature is not employed in our method. In addition, our experiment results show that there is a strong negative correlation between the *V* channel used in [17] and our indicator defined in the next subsection, so we do not use the *V* channel in the proposed method.

As a result, the adjusted saturation feature $f_{satu}$ is defined as


$$f_{satu}=\frac{1}{\omega }\frac{1}{mn}\sum xS(x),$$ (5)

where *m* and *n* are the row and column of matrix *S*, and the adjusted weight ω is defined as


$$\omega =\max_{x}S(x)\cdot \left(1+\alpha e^{{\left(\frac{d}{\beta }\right)}^{2}}\right),$$ (6)

where *d* is the visibility distance; α and β are parameters specified according to experiment. In our experiment, α=0.05,β=20000, and d=500, used as default values, are set according to the parameter adjustment for fog density estimation experiments and the visibility distance with moderate density. The design of adjusting weights is based on an exponential decay feature of visibility.

#### 2.2.3. Constructing the image’s grayscale points diffusion feature.

Inspired by the relationship between the image fog density to the Jdark image [18] and the image grayscale entropy [14], it is a natural idea to use the grayscale information to perceive the fog density of the image. Therefore, we define a grayscale points diffusion feature $f_{rate}$which is a ratio index of grayscale points to characterize fog density information. Given that a pixel of a foggy image often shows a gray color, whether this pixel is fogged up G(x) can be inferred through the following rule.


$$G(x)=\left\{ \begin{array}{c} 0,\sigma (x)\geq \sigma_{g}\\ 1,\sigma (x)<\sigma_{g} \end{array}\right.,$$ (7)

where G(x)=1 means that the pixel *x* is a gray point. $\sigma_{g}$ is a parameter and specified by user or experiment analysis ($Defaultvalue\sigma_{g}=$0.018, which is the result of the experimental test). σ(x) is the RGB variance of the pixel *x*, and it can be calculated by


$$\sigma (x)=\sqrt{\frac{1}{2}\left(x_{R}^{2}+x_{G}^{2}+x_{B}^{2}-\frac{1}{3}{\left(x_{R}+x_{G}+x_{B}\right)}^{2}\right)}.$$ (8)

Obviously, according to Eq (8), if $x_{R}=x_{G}=x_{B}$, then σ(x)=0, i.e., the pixel *x* is a gray point. Using Eq (7), the gray points in the image *I* can be counted and the rate $r_{gray}$ of the gray points to all pixels can be calculated as


$$r_{gray}=\frac{1}{mn}\sum x\in IG(x).$$ (9)

Given that there is a certain percentage $\gamma_{0}$ of gray points in an image, even if the image is fog-free, the gray point diffusion feature $f_{rate}$ can be defined by adjusting $r_{gray}$, as


$$f_{rate}=\frac{r_{gray}-\gamma_{0}}{1-\gamma_{0}}.$$ (10)

Note that $\gamma_{0}$ is specified and meets this condition $\gamma_{0}\leq r_{gray}$. In addition, from another perspective, $f_{rate}$ can be explained as a noise diffusion index. In fact, in the literature on dehazing research, multiple researchers have used fog as noise and built models to dehaze and defog [29–31].

#### 2.2.4. Feature fusion.

The fourth step is feature fusion. These three significant features, $f_{dark}$, $f_{satu}$, and$f_{rate}$, can be weighted and linearly combined to estimate the fog density $f_{den}$ in the image as


$$f_{den}=w_{1}f_{dark}+w_{2}f_{satu}+(1-w_{1}-w_{2})f_{rate},$$ (11)

where $w_{i}(i=1,2)$ is the weight and $0\leq w_{i}\leq 1$,$0\leq w_{1}+w_{2}\leq 1.$ These parameters are specified according to the experiment. For images with large areas of sky or gray-white objects, due to the large error in dark channel calculation results [20,21], their weights can be appropriately reduced, for example $w_{1}=0.10.$ In our experiment, $w_{1}=0.25$ and $w_{2}=0.50$ are used as default values, set according to the parameter adjustment for fog density estimation experiments and the effect of Jdark.

### 2.3. Evaluation index

We have noticed two noteworthy characteristics in some fog density estimation methods and datasets. One characteristic is that some methods estimate fog density values not limited to numbers between 0 and 1, such as GDEn [14] and FADE [15]. The other characteristic is that different synthesized datasets have inconsistent labeling scales for fog density. For example, the CHIC dataset labeled fog density levels from level 1 to level 10 (Fig 1), and the Cityscapes dataset labeled fog density levels from level 0.005 to level 0.02 (Fig 2). These characteristics make it challenging to evaluate the accuracy of various methods when the exact value of fog density is unknown. Assuming that both the order of fog density level labels and the difference between each level in the synthetic dataset are accurate, we propose two indicators, namely the sequential error index $\eta_{ord}$and the ratio error index $\eta_{den}$, to evaluate the accuracy of various image fog density estimation methods.

The sequential error index is used to evaluate whether the estimated density order of the image sequence is consistent with the actual value order. Let $i_{1},i_{2},\cdots ,i_{k},$ be the estimated sequence of image sequence $I_{1},I_{2},\cdots ,I_{k}$. Both sequences are in the order of fog density from fog-free to heavy fog image. The index $\eta_{ord}$ of sequential error can be defined as


$$\eta_{ord}=\frac{1}{k}\sum_{t=1}^{k}{\left(t-i_{t}\right)}^{2}.$$ (12)

Note that the smaller the value of $\eta_{ord}$, the more accurate the fog density estimation result and the smaller the error. When the sequential error index $\eta_{ord}=0$, the density order of the estimated values is consistent with the order of the original actual values.

The ratio error index is used to evaluate whether the ratio of the estimated density values of the image sequence to the true values is stable. Considering that the specified range of estimated values obtained by different algorithms may not necessarily be the same as the defined range of actual values, the consistency here mainly evaluates the consistency of the ratio of estimated density values to actual density values.

Let $f_{den}^{\left(1\right)},f_{den}^{\left(2\right)},\cdots ,f_{den}^{\left(k\right)},$ and $f_{gt}^{\left(1\right)},f_{gt}^{\left(2\right)},\cdots ,f_{gt}^{\left(k\right)}$ be the estimated density values and the ground truth density value of image sequence $I_{1},I_{2},\cdots ,I_{k},$ respectively. The ratio error index $\eta_{den}$ is defined as


$$\eta_{den}=\sqrt{\frac{1}{k}\sum_{j=1}^{k}{\left(r_{den}^{\left(i\right)}-{\overset{―}{r}}_{den}\right)}^{2}},$$ (13)

where $r_{den}^{\left(i\right)}$ is the density ratio of exponential function of the estimated density value and the ground truth density value of image $I_{i}$, i.e.,


$$r_{den}^{\left(i\right)}=\frac{e^{\left|f_{den}^{\left(i\right)}\right|}}{e^{\left|f_{gt}^{\left(i\right)}\right|}}=e^{\left|f_{den}^{\left(i\right)}-f_{gt}^{\left(i\right)}\right|},$$ (14)

and


$${\overset{―}{r}}_{den}=\frac{1}{k}\sum_{j=1}^{k}r_{den}^{\left(i\right)}.$$ (15)

In Eq (14), the exponential function is used to avoid the denominator being zero. When $r_{den}^{\left(i\right)}=0$, the estimated density value is the same as the ground truth density value.

Note that both the sequential error index $\eta_{ord}$and the ratio error index $\eta_{den}$ are better if their values are smaller.

## 3. Experimental results

We use the datasets described in subsection 2.1 to evaluate the proposed method (Ours).

### 3.1. Consistency analysis

We use two synthetic datasets to check if the estimated fog densities match the density values annotated in these datasets (ground truth).

#### 3.1.1. Results of color hazy image database.

**Fig 6:**
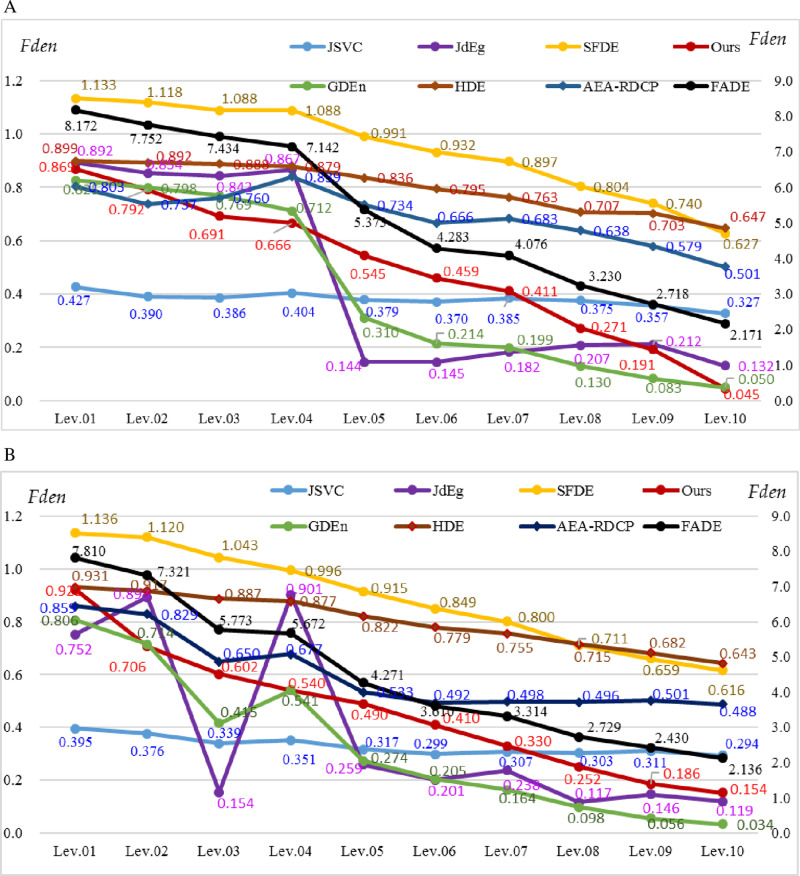
The estimated fog density of ten images from two Scenes in CHIC. From left to right, Lev.01 means heavy fog, and Lev.10 is fog-free. FADE uses the right *y*-axis, and other methods use the left *y*-axis. (a) Scene1, (b) Scene2.


The first experiment data are two scenes in the Color Hazy Image Database (CHIC) [26,27]. All ten images of Scene1 (Fig 1) are labeled with a density from 1 (heavy foggy) to 10 (fog-free). The fog density $f_{den}$ is calculated using our method (named Ours) and compared to the results from GDEn [14], SFDE [16], FADE [15], JSVC[17], JdEg[20], HDE[19], AEA-RDCP[21], and the ground truth, as shown in Fig 6. Noted that the density value estimated using GDEn ranges interval [2,10] and is inversely proportional to the fog density value, so it has been transformed into [0,1] by the formula as

Fden=1−0.125(GDEn−2). (16)

This formula is used for all calculations related to GDEn in the following experiments.

Note that the CHIC database is characterized by the presence of the ground truth reference images as the horizontal axis coordinate display.

According to Fig 6, we can conduct further observation and statistical analysis:

(1) The fog density is represented using different scale numbers by different methods. The large one is 8.172, as FADE estimates, which lacks standardization. A more convenient range for comparison and understanding is [0, 1], where 0 represents fog-free, and 1 means heavy fog, as outputs by methods JdEg, Ours, HDE, AEA-RDCP, and Adjusted GDEn.
(2) We calculate seven statistical indicators: the standard deviation (Std), the average (Ave), the coefficient of variation (CV), mean squared error (MSE), mean absolute error (MAE), t-test value (t-value), and p-value, and list them in Table 2. From the table, it can be found that our method ranks in the middle of the eight methods in terms of Std and CV value of the estimation results; our method gets the minimum values of MSE and MAE, as well as the maximum p-value. According to the p-value, the significance level, and t-value, the difference between the fog density estimation results of these three methods (JSVC, JdEg, and Ours) and the actual values is insignificant, which means that the three methods get the best performance.

**Table 2:** Calculation results comparison of seven statistical indicators of the fog density are estimated using eight different methods for Scene1 (the up seven rows) and Scene2 (the down seven rows).

Index.	JSVC	FADE	JdEg	SFDE	GDEn	HDE	AEA-RDCP	Ours
Std	0.0265	2.2469	0.3593	0.1743	0.3251	0.0921	0.1028	0.2691
Ave	0.3799	5.2352	0.4477	0.9418	0.4090	0.8008	0.6941	0.4939
CV	0.0699	0.4292	0.8026	0.1851	0.7949	0.1150	0.1481	0.5448
MSE(↓)	0.0851	25.8425	0.0358	0.2125	0.0177	0.1318	0.0800	0.0020
MAE(↓)	0.2489	4.7352	0.1437	0.4418	0.1071	0.3111	0.2461	0.0357
t_value	0	1	0	1	1	1	1	0
p_value(↑)	0.2084	0.0000	0.4105	0.0000	0.0205	0.0016	0.0197	0.6893
Std	0.0348	2.0322	0.3304	0.1882	0.2767	0.1016	0.1443	0.2435
Ave	0.3293	4.5066	0.3779	0.8846	0.3306	0.8009	0.6023	0.4593
CV	0.1055	0.4509	0.8741	0.2128	0.8369	0.1268	0.2396	0.5301
MSE(↓)	0.0958	18.7693	0.0641	0.1600	0.0366	0.1273	0.0419	0.0073
MAE(↓)	0.2625	4.0066	0.1947	0.3846	0.1694	0.3046	0.1481	0.0689
t_value	0	1	0	1	1	1	0	0
p_value(↑)	0.0787	0.0000	0.1332	0.0000	0.0003	0.0011	0.1176	0.1381

Note: in the t-test, the significance level is specified as 0.05.

#### 3.1.2. Results of the Cityscapes dataset.

Our experiment has been conducted on the Cityscapes dataset [22,23]. After using different methods to estimate the fog density of each image, the average fog densities of each group have been listed in Table 3. From the column of Ours in the table, we can find that these images have medium to low densities because they are just less than 0.5, consistent with our observation in Fig 2. According to the fog densities estimated by JdEg and GDEn, this fog is very thin (less than 0.2). Overall, the results of JSVC, HDE, AEA-RDCP, and Ours are acceptable from the perspective of visual perception of the images.

**Table 3:** The average fog density of each group was estimated using eight methods.

Den.	JSVC	FADE	JdEg	SFDE	GDEn	HDE	AEA-RDCP	Ours
$G_{0.005}$	0.1591	1.3063	0.0069	0.5526	0.1301	0.4836	0.1358	0.2941
$G_{0.01}$	0.2254	1.6563	0.0281	0.6886	0.1325	0.5494	0.2289	0.3546
$G_{0.02}$	0.3353	2.3418	0.1622	0.8614	0.1439	0.6428	0.3850	0.4331

#### 3.1.3. Analysis with evaluation indexes.

According to Tables 2 and 3, it is difficult to distinguish which method estimates more accurately through traditional statistical indicators such as variance and average of the estimated results. At this point, using the proposed two statistical indicators, the sequential error index $\eta_{ord}$(Eq (12)) and the ratio error index $\eta_{den}$(Eq (13)), is a good alternative strategy.

For the fog densities estimated by these eight methods, the calculation results of the sequential error index $\eta_{ord}$ are shown in Table 4. The table shows that only the fog densities estimated by JSVC, GDEn, AEA-RDCP, and JdEg are not consistent for Scene1 and Scene2. Other methods, FADE, SFDE, and Ours, keep the estimated fog densities in the same order as the input data and get the best performance. For the leftImg8 (from the Cityscapes dataset), only the fog densities estimated by GDEn, AEA-RDCP, and HDE have sequential consistency errors.

**Table 4:** The results of the sequential error index $\eta_{ord}\;$calculated using the densities estimated by eight methods.

Dataset	JSVC	FADE	JdEg	SFDE	GDEn	HDE	AEA-RDCP	Ours
Scene1	1.6	0.0	4.6	0.0	0.0	0.0	1.6	0.0
Scene2	2.0	0.0	4.0	0.0	0.2	0.0	2.0	0.0
leftImg8	0.0	0.0	0.0	0.0	0.88	0.04	0.01	0.0

* leftImg8 is leftImg8Val_lindau1 dataset.

The calculation results of the ratio error index $\eta_{den}$ are shown in Table 5. The table shows that only the fog densities estimated by GDEn and Ours are stable (less than 0.2) for all three datasets in view of the ratio error index.

**Table 5:** The results of the ratio error index $\eta_{den}\;$calculated using the densities estimated by eight methods.

Dataset	JSVC	FADE	JdEg	SFDE	GDEn	HDE	AEA-RDCP	Ours
Scene1	0.241	455.779	0.179	0.188	0.110	0.232	0.145	**0.044**
Scene2	0.261	295.978	0.250	0.154	0.113	0.230	0.141	**0.067**
leftImg8	0.135	7.095	0.160	0.449	**0.057**	0.154	0.204	0.170

By comparing Tables 4 and 5, we can safely conclude that the estimated fog densities using our method (Ours) keep the order along the input image fog density and have the minimum ratio error.

### 3.2. Real road fog density analysis

**Fig 7:**
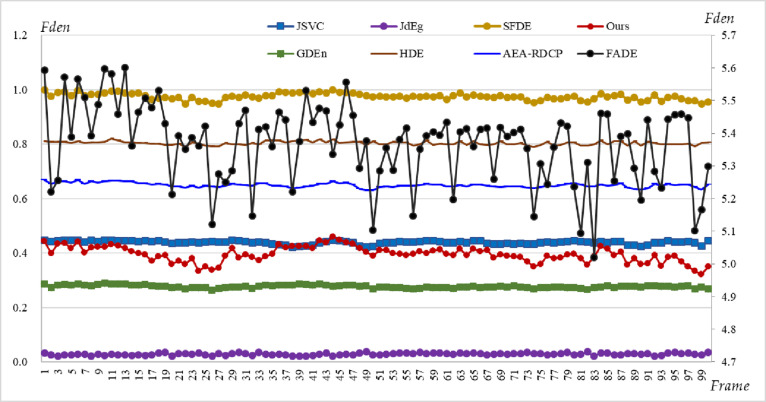
The fog densities of 100 keyframes of highway monitoring estimated using eight methods. The FADE line uses the right *y*-axis, and the other lines use the left *y*-axis.

For the 100 keyframes of highway monitoring (Fig 3), we use eight methods, JSVC, FADE, JdEg, SFDE, GDEn, HDE, AEA-RDCP, and Ours, to estimate the fog density of each frame, and the estimated fog density of each frame is shown in Fig 7. From the figure, it can be found that the line charts drawn from the estimation results of these eight methods are relatively stable; the estimated values of SFDE and HDE methods are greater than 0.8, which means that this is a scene with heavy fog; the estimated values of both JdEg and GDEn methods are less than 0.3, which means that this is a scene with thin fog. The last two statements contradict each other. However, compared to Fig 3, it can be seen that the fog density is moderate, which is consistent with the density values estimated by Ours, AEA-RDCP, and JSVC. Note that since the estimated values of the FADE method are not standardized to the range of 0–1, its estimation results are suitable for relative comparison, but not for directly indicating the fog density based on the estimated values.

Although the eight estimation curves in Fig 7 are relatively stable, the fluctuations in the estimation curves of both FADE and Ours methods are relatively large, which can also be proved by their standard variances (Marked as Std in Table 6).

**Table 6:** Standard variances comparison and trend analysis by linearly fitting using eight methods.

Method	JSVC	FADE	JdEg	SFDE	GDEn	HDE	AEA-RDCP	Ours
Std	0.0065	0.1222	0.0041	0.0120	0.0053	0.0068	0.0081	0.0285
Intercept	0.4417	5.4435	0.0264	0.9838	0.2804	0.8062	0.6559	0.4154
Slope($\times 10^{-2}$)	-0.0062	-0.1482	0.0039	-0.0180	-0.0076	-0.0054	-0.0130	-0.0398
$R^{2}$	0.0776	0.1225	0.0740	0.1859	0.1692	0.0536	0.2149	0.1619
p-value	0.0050	0.0004	0.0062	0.0000	0.0000	0.0205	0.0000	0.0000

**Fig 8:**
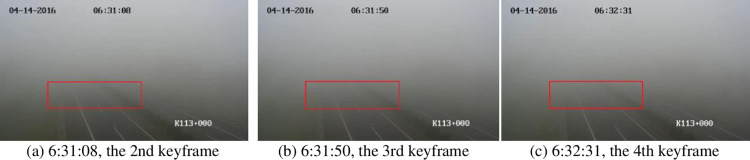
The second, third, and fourth keyframes of the highway monitoring video. By comparing the lane lines in the red rectangular areas of the three subgraphs, we can see (Zoom in, please) that the fog density in subgraph (a) is significantly lower than that in subgraphs (b) and **(c)**, and also lower than that in **Fig. 3(a)**. This is consistent with the values in **Fig 7**.

Due to the lack of actual fog density values, the accuracy of the estimation results needs to be subjectively evaluated through intuitive observation. The second, third, and fourth keyframes are displayed in Fig 8 because the fog density changes significantly in these three frames by referring to the results of FADE. Comparing the lane lines in the red rectangular regions of the keyframes, we can find that the fog density in Fig 8(a) is slightly lighter than the fog in Fig 8(b) and Fig 8(c); the difference in fog density between Fig 8(b) and Fig 8(c) is relatively small. These two observation conclusions are consistent with our quantitative calculation results (red line in Fig 7).

A practical task is to infer the dissipation time of fog based on the estimated fog density. We here conduct a trend analysis. By linearly fitting the estimated results of each method, the intercept (*b*), slope (*k*), $R^{2}$, and p-value of the fitted line can be calculated (Listed in Table 6). Although all values of $R^{2}$ are less than 0.25, indicating that all linear models are not significant, all p-values are less than the significance level 0.05, indicating that all linear models are acceptable. In addition, if slope k>0, it indicates that the fog will gradually become thicker; If k<0, it indicates that the fog will gradually dissipate; If k=0, it can be considered that the density of fog has not changed significantly. From Table 6, it can be found that except for the calculation results of method JdEg, the slopes calculated by the other seven methods are all negative, which means that over time, the density of fog gradually decreases and eventually dissipates. This trend inference is consistent with our intuitive perception of the data (Fig 3).

If we employ the formula $t=(f_{den}-f_{0})/k$, and let $f_{0}=0.2$ which means that the fog will dissipate, the time of the fog will last 44.41, 40.54, 51.50, 49.95, 12.18, 128.0, 40.0, and 6.21 hours starting from the timer (6:30:26) according to the results of eight methods, JSVC, FADE, JdEg, SFDE, GDEn, HDE, AEA-RDCP, and Ours respectively. Our method predicts that the fog will dissipate after 12 o’clock on a cloudy day, which seems to be a possible phenomenon. Unfortunately, no error analysis was conducted here due to the lack of actual time data.

### 3.3. Real airport video fog density analysis

**Fig 9:**
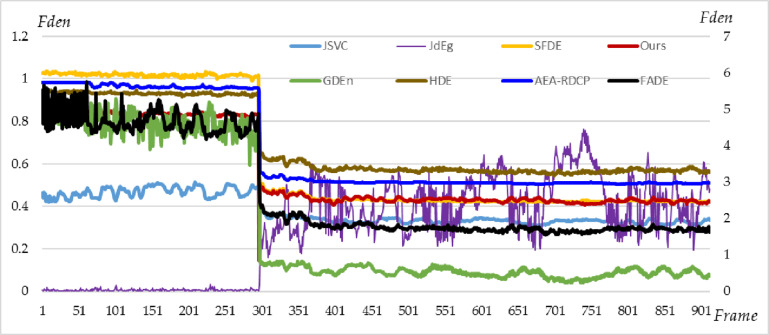
Eight methods are used to estimate the fog density of the airport monitoring video’s 912 keyframes. The line of FADE uses the right *y*-axis, and the other lines use the left *y*-axis.

For the airport monitoring video (Fig 4), we use eight methods, JSVC, FADE, JdEg, SFDE, GDEn, HDE, AEA-RDCP, and Ours, to estimate the fog density of 912 keyframes captured from the video. The estimated fog density of each keyframes is shown in Fig 9. From the figure, we can find that JdEg provides an estimated fog density curve with an upward trend, which does not match the actual observation results of Fig 4; the result calculated using JSVC is relatively stable; the curve shape drawn by Ours, SFDE, GDEn, HDE, AEA-RDCP, and FADE is quite similar.

**Fig 10:**
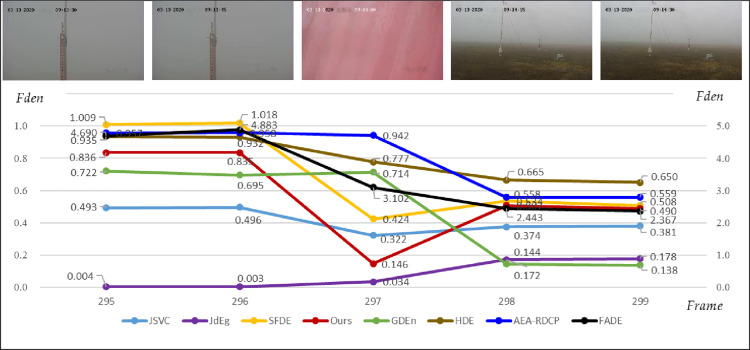
The keyframes from the 295^th^ to the 299^th^ and their estimated fog density values estimated using eight methods.

Given that there is a notable change in the fog density estimates before frame 301 for each method in Fig 9, we display the actual images from the 295th to the 299th frames and their fog density values estimated using eight methods, as shown in Fig 10. In this short period (from 9:13:30–9:14:30), the camera’s azimuth angle was adjusted, and there was shaking, so the captured image scenes were different, and the fog density in the image also changed significantly. From the perspective of reflecting the fog density of the image, the calculation results of methods Ours and SFDE can well reflect this drastic change. These results show that our method and SFDE have the best sensitivity to image fog density.

Table 7 lists several statistical indicators of fog density estimation results calculated by those eight methods for the airport video keyframes. By comparing the numbers in the table, our results best match the density of fog in actual videos according to the maximum, minimum, and average values. Our method estimates a maximum value of 0.849, a minimum value of 0.146, and an average of 0.562, which means that the fog in this scene gradually changes from heavy fog to thin fog, with an average of medium fog. However, according to the results of FADE, the fog density level cannot be determined; according to the results of JSVC, the fog density level is medium or light fog; according to the minimum values of JdEg and GDEn, the fog density level is fog-free; according to the minimum values of HDE and AEA-RDCP, the fog density level is always heavy.

**Table 7:** The descriptive statistics analysis of fog density estimated using eight methods.

	JSVC	FADE	JdEg	SFDE	GDEn	HDE	AEA-RDCP	Ours
Max	0.515	5.760	0.764	1.036	0.912	0.947	0.984	0.849
Min	0.309	1.553	0.001	0.416	0.040	0.545	0.503	0.146
Ave	0.377	2.711	0.287	0.621	0.320	0.690	0.661	0.562
Std	0.066	1.389	0.223	0.276	0.330	0.168	0.213	0.190

### 3.4. Time efficiency

All experiments are conducted on a personal laptop. The hardware parameters are the 11th Gen Intel(R) Core(TM) i7-11800H @ 2.30GHz 2.30 GHz, with 44G RAM and 64-bit OS. The software environment is Windows 11, and coding with MATLAB. The running time of estimating fog density using eight methods in all four datasets, Scene1 (10 images), Scene2(10 images), leftImg8Val_lindau1(171 images), the Key Frames of Highway (100 images), are listed in Table 8. Among the eight methods, SFDE goes the fastest; GDEn and AEA-RDCP are the most time-consuming. Our method has a similar time cost to the other four methods because of calculating Jdark map.

**Table 8:** The average time (Unit: s) consumption using eight methods to estimate various datasets.

Dataset	JSVC	FADE	JdEg	SFDE	GDEn	HDE	AEA-RDCP	Ours
Scene1	19.43	24.44	17.86	3.82	178.33	12.53	295.27	17.68
Scene2	19.38	24.02	18.05	3.80	163.13	4.64	238.61	17.57
leftImg8	342.35	409.59	313.48	64.39	2744.01	98.68	4575.80	317.22
Highway Frames	82.89	104.57	76.47	15.86	679.41	18.33	710.86	76.43
Airport Video	808.18	1053.66	729.61	152.84	8800.61	180.55	7661.87	786.47

* Highway Frames is the Key Frames of Highway dataset.

## 4. Discussion

To check if the three features used in our method are redundant, we sequentially remove one of them and derive three methods: JdSa, JdGR, and SaGR.

JdSa does not use the number of gray points information, and is built using two features, $f_{dark}$, $f_{satu}$. Its density estimation formula is


$$f_{JdSa}=\alpha_{1}f_{dark}+\left(1-\alpha_{1}\right)f_{satu}.$$ (17)

JdGR does not use the saturation information, and is built using two features, $f_{dark}$, $f_{rate}$. Its density estimation formula is


$$f_{JdGR}=\beta_{1}f_{dark}+(1-\beta_{1})f_{rate}.$$ (18)

SaGR does not use the dark channel map, and is built using two features, $f_{satu}$, $f_{rate}$. Its density estimation formula is


$$f_{SaGR}=\gamma_{1}f_{satu}+(1-\gamma_{1})f_{rate},$$ (19)

where parameters $\alpha_{1}=0.25,\beta_{1}=0.25$, and $\gamma_{1}=0.5$ are set as default values according to the parameter tuning in our experiments.

**Fig 11:**
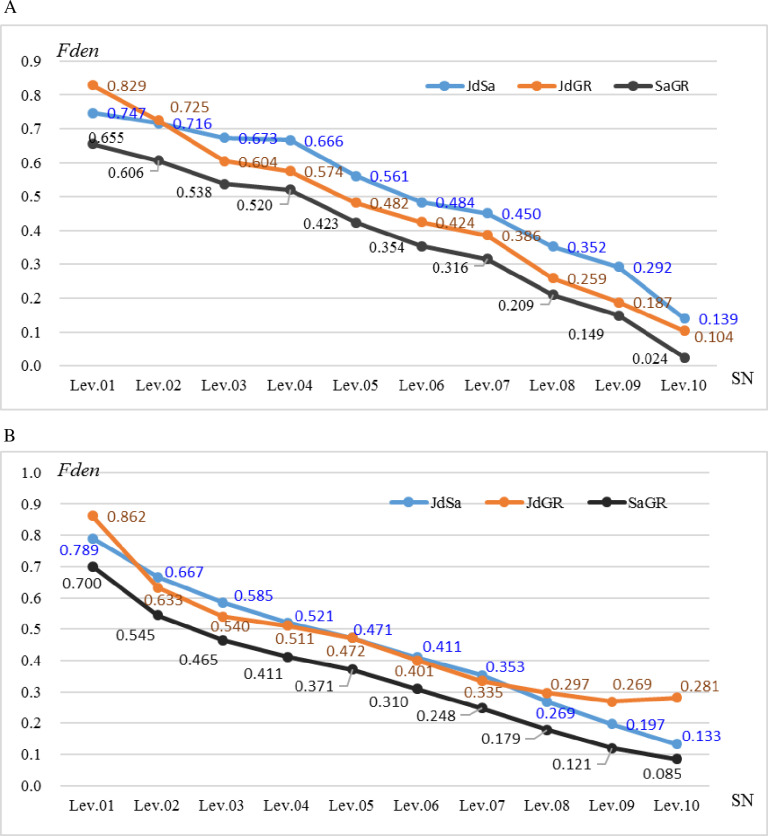
The estimated fog density of ten images from two Scenes in CHIC using three methods: JdSa, JdGR, and SaGR. From left to right, Lev.01 means heavy fog, and Lev.10 is fog-free. These methods use two features among $f_{dark}$, $f_{satu}$, $f_{rate}$. (a) Scene1, (b) Scene2.

For convenient comparison, we use the CHIC dataset to test. The fog densities estimated using JdSa, JdGR, and SaGR are drawn as three lines in Fig 11. This figure shows that all three lines are monotonically decreasing, which means all three methods can achieve image-based fog density estimation.

Then, we use the proposed two evaluation indexes $\eta_{ord}$ and $\eta_{den}$ to evaluate JdSa, JdGR, and SaGR. The calculation results of these three methods and the proposed method (Ours) are listed in Table 9. By checking the table, we can find that the JdGR method cannot maintain sequential consistency; and the SaGR method, without Jdark information, shows the largest ratio error index among the four methods. Overall, based on the experimental results, the proposed method (Ours) performs better than any of the other three methods in view of $\eta_{den}$. Therefore, directly removing any feature from the proposed methods will cause a decrease in the accuracy of fog density estimation.

**Table 9:** Two evaluation indexes $\eta_{ord}$ and $\eta_{den}\;$ are calculated using the densities estimated by four methods.

	$\eta_{ord}$				$\eta_{den}$			
Dataset	JdSa	JdGR	SaGR	Ours	JdSa	JdGR	SaGR	Ours
Scene1	0	0	0	0	0.084	0.077	0.116	0.044
Scene2	0	0.2	0	0	0.094	0.094	0.132	0.067

## 5. Conclusions

To estimate the fog density based on an image, we in this paper propose a method (JSFD) that integrates three features: the image saturation, the dark channel map, and the degree of fog diffusion. The proposed method demonstrates the following three advantages: Firstly, the fog density range output by our method falls into [0,1], which is in line with general understanding and facilitates comparison of fog density in different scenarios; Secondly, the fog density output by the proposed method has good consistency with the synthetic fog image datasets; Thirdly, actual highway monitoring video and the airport monitoring video data show that the new method can reflect the changes in fog density in these transport scenes. In addition, we define two indexes, the sequential error index and the ratio error index, which can effectively measure whether a fog density estimation method is effective.

However, there are still some limitations to the proposed method. First, due to the lack of actual fog density values and the limited number of open image sets with accurate density labeling, the accuracy testing, including the sequential error index, and the ratio error index, is not yet sufficient and more experiments are needed. Second, if visibility data can be collected during video surveillance, it can be used to compare and calibrate the fog density estimation results, referring to the existing approach [32]. Third, referring to the 297^th^ frame in Fig 10, environmental factors such as lighting and shadows may interfere with the accuracy of estimating fog density. Therefore, combining these factors to estimate fog density is a topic worth studying, as it can increase the practicality of estimating fog density in different weather conditions. In addition, the use of deep learning and large models for continuous value estimation of fog density is also worth further research in the future.

## References

[1] Hagiwara T, Yagi M, Seo T. Visibility of Laser beams and illuminated delineator as a function of fog density. Transport Res Record: J Transport Res Board. 1996;1553(1):59–65. doi: 10.1177/0361198196155300109

[2] Mori K, Takahashi T, Ide I. Fog density recognition by in-vehicle camera and millimeter wave radar. Int J Innov Comput Inf Control. 2007;3(5):1173–82.

[3] Liu PJ. Enhance low visibility image using haze-removal framework. IEEE Access. 2023;11:113450–63. doi: 10.1109/access.2023.3322041

[4] Nadeem F, Leitgeb E. Dense maritime fog attenuation prediction from measured visibility data. Radioengineering. 2010, 19(2): 223–7.

[5] Ovseník L, Turán J, Mišenčík P, et al. Fog density measuring system. Acta Electrotechnica et Informatica. 2012, 12(2): 67–71. doi: 10.2478/v10198-012-0021-7

[6] Ovsenik L, Turan J, Tatarko M, Turan M, Vasarhelyi J. Fog sensor system: Design and measurement. In: Proceedings of the 13th International Carpathian Control Conference (ICCC). IEEE. 2012. 529–32. doi: 10.1109/carpathiancc.2012.6228701

[7] Negru M, Nedevschi S. Image based fog detection and visibility estimation for driving assistance systems. 2013 IEEE 9th International Conference on Intelligent Computer Communication and Processing (ICCP), Cluj-Napoca, Romania. 2013: 163–168.

[8] Hautiére N, Tarel J-P, Lavenant J, Aubert D. Automatic fog detection and estimation of visibility distance through use of an onboard camera. Machine Vision and Applications. 2006;17(1):8–20. doi: 10.1007/s00138-005-0011-1

[9] Anwar MdI, Khosla A. Fog Classification and Accuracy Measurement Using SVM. In: 2018 First International Conference on Secure Cyber Computing and Communication (ICSCCC). IEEE. 2018. 198–202. doi: 10.1109/icsccc.2018.8703365

[10] Chen Y, Wang J, Li S, Wang W. Multi-feature based foggy image classification. IOP Conf Ser: Earth Environ Sci. 2019;234:012089. doi: 10.1088/1755-1315/234/1/012089

[11] Dong J, Cao X, Zhou S, Zhao S, Zhang D. Image Fog Density Recognition Method Based on Multi-Feature Model and S-DAGSVM. In: 2020 International Conference on Artificial Intelligence and Electromechanical Automation (AIEA). IEEE. 2020. 1–6. doi: 10.1109/aiea51086.2020.00008

[12] Yang W, Zhao Y, Li Q, Zhu F, Su Y. Multi visual feature fusion based fog visibility estimation for expressway surveillance using deep learning network. Expert Systems with Applications. 2023;234:121151. doi: 10.1016/j.eswa.2023.121151

[13] Caraffa L, Tarel JP. Daytime fog detection and density estimation with entropy minimization. ISPRS Ann Photogramm Remote Sens Spatial Inf Sci. 2014;II–3:25–31. doi: 10.5194/isprsannals-ii-3-25-2014

[14] Cao R, Wang X, Li H. Fog density evaluation by combining image grayscale entropy and directional entropy. Atmosphere. 2023;14(7):1125. doi: 10.3390/atmos14071125

[15] Choi LK, You J, Bovik AC. Referenceless prediction of perceptual fog density and perceptual image defogging. IEEE Trans Image Process. 2015;24(11):3888–901. doi: 10.1109/TIP.2015.2456502 26186784

[16] Ling Z, Gong J, Fan G, Lu X. Optimal transmission estimation via fog density perception for efficient single image defogging. IEEE Trans Multimedia. 2018;20(7):1699–711. doi: 10.1109/tmm.2017.2778565

[17] Jiang Y, Sun C, Zhao Y, Yang L. Fog density estimation and image defogging based on surrogate modeling for optical depth. IEEE Trans Image Process. 2017;26(7):3397–409. doi: 10.1109/TIP.2017.2700720 28475053

[18] He K, Sun J, Tang X. Single image haze removal using dark channel prior. IEEE Trans Pattern Anal Mach Intell. 2011;33(12):2341–53. doi: 10.1109/TPAMI.2010.168 20820075

[19] Ngo D, Lee G-D, Kang B. Haziness degree evaluator: a knowledge-driven approach for haze density estimation. Sensors (Basel). 2021;21(11):3896. doi: 10.3390/s21113896 34200061 PMC8200195

[20] Guo H, Wang X, Li H. Density estimation of fog in image based on dark channel prior. Atmosphere. 2022;13(5):710. doi: 10.3390/atmos13050710

[21] Hwang S-H, Kwon K-W, Im T-H. AEA-RDCP: an optimized real-time algorithm for sea fog intensity and visibility estimation. Applied Sciences. 2024;14(17):8033. doi: 10.3390/app14178033

[22] Cordts M, Omran M, Ramos S. The cityscapes dataset for semantic urban scene understanding. In: Proceedings of the IEEE Conference on Computer Vision and Pattern Recognition. IEEE. 2016. 3213–23.

[23] McMillan J, Bester MJ, Apostolides Z. In silico docking and ADMET studies on clinical targets for type 2 diabetes correlated to in vitro inhibition of pancreatic alpha-amylase and alpha-glucosidase by rutin, caffeic acid, p-coumaric acid, and vanillin. In Silico Pharmacol. 2025;13(1):42. doi: 10.1007/s40203-025-00324-6 40093583 PMC11906964

[24] Xie Y, Wei H, Liu Z, et al. SynFog: a photo-realistic synthetic fog dataset based on end-to-end imaging simulation for advancing real-world defogging in autonomous driving. In Proceedings of the IEEE/CVF Conference on Computer Vision and Pattern Recognition (CVPR). 2024: 21763–21772. doi: 10.1109/CVPR52733.2024.02056

[25] Miclea R-C, Ungureanu V-I, Sandru F-D, Silea I. Visibility enhancement and fog detection: solutions presented in recent scientific papers with potential for application to mobile systems. Sensors (Basel). 2021;21(10):3370. doi: 10.3390/s21103370 34066176 PMC8150865

[26] El Khoury J, Thomas J-B, Mansouri A. A database with reference for image dehazing evaluation. J Imaging Sci Technol. 2018;62(1):10503–13. doi: 10.2352/j.imagingsci.technol.2018.62.1.010503

[27] Zhao W, Sun X, Wu S, Wu S, Hu C, Huo H, et al. MaGA20ox2f, an OsSD1 homolog, regulates flowering time and fruit yield in banana. Mol Breed. 2025;45(1):12. doi: 10.1007/s11032-024-01523-3 39803631 PMC11717755

[28] Androutsos D, Plataniotis KN, Venetsanopoulos AN. A novel vector-based approach to color image retrieval using a vector angular-based distance measure. Computer Vision and Image Understanding. 1999;75(1–2):46–58. doi: 10.1006/cviu.1999.0767

[29] Tripathi AK, Mukhopadhyay S. Single image fog removal using anisotropic diffusion. IET Image Process. 2012;6(7):966–75. doi: 10.1049/iet-ipr.2011.0472

[30] Gibson KB, Nguyen TQ. Fast single image fog removal using the adaptive Wiener filter. In: 2013 IEEE International Conference on Image Processing. IEEE. 2013. 714–8. doi: 10.1109/icip.2013.6738147

[31] Li W, Zhou G, Wang X. Low illumination fog noise image denoising method based on ACE-GPM. PLoS One. 2024;19(5):e0302492. doi: 10.1371/journal.pone.0302492 38713661 PMC11075849

[32] Dai M, Li G, Shi W. Fog density analysis based on the alignment of an airport video and visibility data. Sensors (Basel). 2024;24(18):5930. doi: 10.3390/s24185930 39338675 PMC11435703

